# Gene Fusion Detection in NSCLC Routine Clinical Practice: Targeted-NGS or FISH?

**DOI:** 10.3390/cells12081135

**Published:** 2023-04-11

**Authors:** Lorenza Pecciarini, Emanuela Brunetto, Greta Grassini, Valeria De Pascali, Francesca Rita Ogliari, Anna Talarico, Giovanna Marra, Gilda Magliacane, Miriam Redegalli, Gianluigi Arrigoni, Chiara Lazzari, Vanesa Gregorc, Alessandra Bulotta, Claudio Doglioni, Maria Giulia Cangi

**Affiliations:** 1 Pathology Unit, IRCCS San Raffaele Scientific Institute, 20132 Milan, Italy; 2 Department of Oncology, IRCCS San Raffaele Scientific Institute, 20132 Milan, Italy; 3 Candiolo Cancer Institute, FPO-IRCCS, 10060 Turin, Italy

**Keywords:** fusion, NGS, FISH, ALK, ROS1, RET, NTRK, molecular testing, NSCLC, lung cancer

## Abstract

The ability to identify the broadest range of targetable gene fusions is crucial to facilitate personalized therapy selection for advanced lung adenocarcinoma (LuADs) patients harboring targetable receptor tyrosine kinase (RTK) genomic alterations. In order to evaluate the most effective testing approach for LuAD targetable gene fusion detection, we analyzed 210 NSCLC selected clinical samples, comparing in situ (Fluorescence In Situ Hybridization, FISH, and ImmunoHistoChemistry, IHC) and molecular (targeted RNA Next-Generation Sequencing, NGS, and RealTime-PCR, RT-PCR) approaches. The overall concordance among these methods was high (>90%), and targeted RNA NGS was confirmed to be the most efficient technique for gene fusion identification in clinical practice, allowing the simultaneous analysis of a large set of genomic rearrangements at the RNA level. However, we observed that FISH was useful to detect targetable fusions in those samples with inadequate tissue material for molecular testing as well as in those few cases whose fusions were not identified by the RNA NGS panel. We conclude that the targeted RNA NGS analysis of LuADs allows accurate RTK fusion detection; nevertheless, standard methods such as FISH should not be dismissed, as they can crucially contribute to the completion of the molecular characterization of LuADs and, most importantly, the identification of patients as candidates for targeted therapies.

## 1. Introduction

Gene fusion detection is crucial in the routine clinical practice of non-small cell lung cancer (NSCLC). While for early and locally advanced NSCLC, only *EGFR* mutational status is mandatory and *ALK* fusion analysis is optional, in metastatic NSCLC, *ALK, ROS1*, *RET* and *NTRK* rearrangements must be investigated together with *EGFR*, *KRAS*, *BRAF*, *HER2* and *MET* mutations in order to identify those oncogene-addicted tumors that can benefit from targeted therapies. Indeed, as recommended by international guidelines, specific tyrosine kinase inhibitors (TKIs) are routinely used for stage IV tumors that carry a rearrangement in the *ALK*, *ROS1*, *NTRK* or *RET* (RTKs) gene [1,2].

Therefore, a main goal for molecular diagnostics in pathology is the ability to identify the broadest range of targetable gene fusions in a short turnaround time in order to facilitate personalized therapy selection for NSCLC patients harboring one of these genomic alterations [3].

It has been reported that 2–7% of lung adenocarcinomas (LuADs) have oncogenic *ALK* rearrangements, which cause the constitutive ligand-independent activation of ALK and the overexpression of the gene by fusing the intact kinase domain of ALK to the N-terminal regions of several different gene partners. *EML4* has been found to be the most prevalent gene fusion partner, but more than 20 other ones have also been identified [4].

*ROS1* and *RET* alterations occur in 1–2% and 1–3% of LuADs, respectively. ROS1 and RET are tyrosine kinase receptors, and similarly to ALK, they undergo genomic rearrangements resulting in fusion proteins that preserve and constitutively activate the tyrosine kinase. The kinase domains of *ROS1* and *RET* are paired with a wide range of partners, the most common being *CD74* for *ROS1* and *KIF5B* for *RET* [4]. 

In all solid tumor types, including LuADs, *NTRK*-gene (*NTRK1*, *NTRK2* and *NTRK3*) alterations have a very low prevalence (0.2–0.4%). NTRK1/2/3 gene alterations are mainly gene fusions, and a wide variety of gene partners has been reported. All of the described fusions result in the constitutive activation of their encoded tropomyosin receptor kinases (TRKA, B and C) and, as a consequence, in the dysregulated activation of the downstream pathways [5]. 

Because RTK gene fusions are mostly caused by chromosomal translocations and intra-chromosomal rearrangements, FISH using break-apart probes has been considered the gold standard method of detection for a long time [2,6]. Since RTK gene fusions result in increased mRNA and protein levels, RT-PCR and IHC have also been successfully used in consideration of their high sensitivity and cost-effectiveness [7]. In particular, positive ALK IHC using an appropriately validated assay may be used to prescribe ALK inhibitors [2].

It is important to underline that standard methods such as IHC, FISH and RT-PCR, traditionally used to investigate the presence of gene rearrangements, have the limitation of examining only one alteration in a single test; therefore, they require more material for all the analyses, resulting in a longer time for complete molecular characterization. Moreover, FISH cannot detect small intrachromosomal rearrangements as the interphase FISH analytical resolution is 100–200 kb, which limits its sensitivity [8].

Given the significant survival benefit obtained from targeted therapies in NSCLC patients with *ALK*, *ROS1*, *RET*, *NTRK1*, *NTRK2* and *NTRK3* gene rearrangements, the identification of these patients should be more and more comprehensive, cost-effective and time-efficient. In the past five years, there has been a growing awareness of the need to move from highly selective testing methods, such as the gold standard FISH analysis, toward a multiplex testing approach that avoids sequential analysis, requires a smaller amount of sample material and saves time.

Next-generation sequencing (NGS) has been demonstrated to be an efficient strategy to address these issues [9,10], allowing the simultaneous analysis of a large set of genomic alterations, including additional clinically relevant RNA alterations such as the exon 14skipped isoform of *MET* and giving a more accurate molecular characterization of each analyzed tumor. Moreover, unlike FISH, NGS data can be used to confirm that the chimeric transcript is in frame and therefore constitutively expressed. 

In order to evaluate the most efficient and cost-effective testing approach for the simultaneous detection of NSCLC gene fusions at the RNA level, in the period 2015–2021, we analyzed 210 NSCLC selected clinical samples using two commercially available RNA NGS-targeted panels and compared the results to those obtained by standard methods (FISH, IHC and RT-PCR). We also collected follow-up clinical data for selected patients who tested positive for an *ALK*, *ROS1* or *RET* fusion and who were treated with targeted therapies at our institution.

## 2. Materials and Methods

### 2.1. Study Cohort

From our molecular diagnostics database, we selected a total of 210 NSCLC cases of lung adenocarcinoma (LuAD) stage IV that were diagnosed at our institution in the period 2015–2021 and for which there was enough tissue material to conduct additional molecular studies. The cohort included 126 males (60%) and 84 females (40%). The patients’ median age at diagnosis was 68 years, ranging from 34 to 89 years. The samples included both surgical samples and tissue biopsies and were reviewed by two expert pathologists (G.A. and C.D.). The histologic diagnosis of lung adenocarcinoma was confirmed for all of them, and for all the specimens, the percentage of the tumor component was >50%.

All the cases included in this study had been analyzed for *EGFR*, *KRAS* and *BRAF* mutations using either a mass spectrometry multiplexed genotyping platform as previously described [11], or a targeted NGS approach [12], and they all resulted in wild type for the mentioned genes. All information regarding human material was managed using anonymous numerical codes, and all samples were handled in compliance with the Declaration of Helsinki (https://www.wma.net/policies-post/wma-declaration-of-helsinki-ethical-principles-for-medical-research-involving-human-subjects/, last accessed on 16 January 2023).

### 2.2. FISH Analysis 

All 210 cases were successfully analyzed by FISH for *ALK*, *ROS1*, *RET*, *NTRK1*, *NTRK2* and *NTRK3*. *ALK*, *ROS1*, *RET*, *NTRK1*, *NTRK2* and *NTRK3* rearrangements were tested by a break-apart FISH probe approach: the ZytoLight SPEC ALK/EML4 TriCheck Probe, ZytoLight SPEC ROS1 Dual Color Break Apart Probe, ZytoLight SPEC RET Dual Break Apart Probe, ZytoLight SPEC NTRK1 Dual Break Apart Probe, ZytoLight SPEC NTRK2 Dual Break Apart Probe and ZytoLight SPEC NTRK3 Dual Break Apart Probe (Zytovision GmbH, Bremerhaven, Germany) were used, following the manufacturer’s suggested protocol. FISH testing was performed on formalin-fixed, paraffin-embedded (FFPE) tissue, utilizing 4μm tumor tissue sections of LuAD specimens as previously described [13]. For each case, a minimum of 50 tumor nuclei were observed independently by two expert cytogenetists using a Zeiss Axioscope system (Zeiss, Milan, Italy), and the analysis was performed using Metafer v4.1.1 software, which is able to count probe signals and identify the number of fused or split signals (MetaSystems s.r.l., Milan, Italy). A sample was called true positive if  ≥15% of tumor nuclei showed convincing split signals (green and red signal distance ≥ 2 signal diameters) or a single 3′ signal, according to international guidelines [2,6]. The presence of an *EML4::ALK* fusion was indicated by green and red split signals, each of which fused to a blue signal in ≥15% of observed tumor nuclei.

### 2.3. ALK IHC

All 210 cases included in the study were analyzed for ALK protein expression by immunohistochemistry (IHC). IHC was performed on FFPE sections using VENTANA ALK (Clone D5F3) CDx Kit and Benchmark Ultra Immunostainer (Ventana Medical Systems, Tucson, AZ, USA) with OptiView detection kit (Ventana Medical Systems, Tucson, AZ, USA), according to the manufacturer’s instructions. The stained sections were analyzed by an expert pathologist (G.A. or C.D.). Presence of strong granular cytoplasmic staining in tumor cells (any percentage of positive tumor cells) was considered positive for ALK, while absence of strong granular cytoplasmic staining in tumor cells was considered negative for ALK. 

### 2.4. RNA Extraction

In all the cases, tumor-rich areas (>50%) were selected by the pathologist in order to perform manual macrodissection prior to total RNA extraction. Total RNA was extracted from all 210 FFPE samples by using the Maxwell RSC RNA FFPE Kit and the Maxwell RSC Instrument (Promega, Milan, Italy), according to the manufacturer’s instructions. Elution was performed in 50 μL and RNA was quantified using the Qubit RNA HS Assay Kit on Qubit 3.0 Fluorometer (ThermoFisher Scientific, Waltham, MA, USA).

### 2.5. Targeted RNA-Based NGS

A total of 94 cases were analyzed by the Oncomine Focus Assay (OFA) RNA Panel (ThermoFisher Scientific, Waltham, MA, USA), and 116 cases were analyzed by the Oncomine Comprehensive Assay v3 (OCAv3) RNA Panel (ThermoFisher Scientific, Waltham, MA, USA). OFA Panel is designed to detect gene fusions involving 23 fusion drivers (*ABL1*, *ALK*, *AKT3*, *AXL*, *BRAF*, *EGFR*, *ERBB2*, *ERG*, *ETV1*, *ETV4*, *ETV5*, *FGFR1*, *FGFR2*, *FGFR3*, *MET*, *NTRK1*, *NTRK2*, *NTRK3*, *PDGFRA*, *PPARG*, *RAF1*, *RET* and *ROS1*). OCAv3 includes the analysis of 51 genes for fusion detection (*AKT2*, *ALK*, *AR*, *AXL*, *BRCA1*, *BRCA2*, *BRAF*, *CDKN2A*, *EGFR*, *ERBB2*, *ERBB4*, *ERG*, *ESR1*, *ETV1*, *ETV4*, *ETV5*, *FGFR1*, *FGFR2*, *FGFR3*, *FGR*, *FLT3*, *JAK2*, *KRAS*, *MDM4*, *MET*, *MYB*, *MYBL1*, *NF1*, *NOTCH1*, *NOTCH4*, *NRG1*, *NTRK1*, *NTRK2*, *NTRK3*, *NUTM1*, *PDGFRA*, *PDGFRB*, *PIK3CA*, *PRKACA*, *PRKACB*, *PTEN*, *PPARG*, *RAD51B*, *RAF1*, *RB1*, *RELA*, *RET*, *ROS1*, *RSPO2*, *RSPO3* and *TERT*). Targeted libraries were prepared either manually or by using the Ion Chef Instrument (ThermoFisher Scientific, Waltham, MA, USA), following the user guide instructions, and then they were sequenced on the 530™ Chip utilizing the S5 Prime sequencer (ThermoFisher Scientific, Waltham, MA, USA). Raw data analysis was performed using Torrent Suite v5.12 (ThermoFisher Scientific, Waltham, MA, USA), and a fusion with a known partner was called present by the Ion Reporter if there were more than 20 supporting reads. 

### 2.6. Real-Time PCR 

*ALK*, *ROS1*, *RET*, *NTRK1*, *NTRK2* and *NTRK3* gene fusions and a *MET* exon 14 skipping alteration were evaluated in 198 cases using the AmoyDx Lung Cancer PCR Panel (Amoy Diagnostics, Xiamen, China), according to the manufacturer’s instructions. The assays were carried out using the SLAN Real-time PCR System (YanengBIO, Shenzhen, China). This panel enables the analysis of 167 hotspot alterations in 11 genes (*EGFR*, *ALK*, *ROS1*, *KRAS*, *BRAF*, *HER2*, *RET*, *MET*, *NTRK1*, *NTRK2* and *NTRK3*) involved in NSCLC at both DNA level for driver mutations and RNA level for gene fusions. RT-PCR results were interpreted in accordance with the kit’s manual. 

## 3. Results

### 3.1. Fusion Variant Detection by FISH and IHC: In Situ Approach

**Figure 1:**
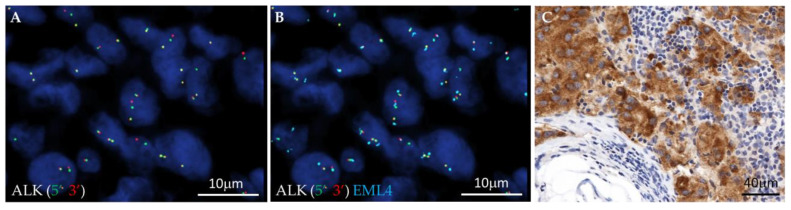
Images showing an example of an *EML4::ALK* rearranged case. FISH analysis shows *ALK* red-green split signals (**A**) fused with *EML4* aqua signals (**B**), and IHC staining shows strong cytoplasmic immunoreactivity (**C**).

A total of 77 cases of the 210 successfully investigated by FISH showed a positive result for *ALK*, *ROS1*, *RET*, *NTRK1*, *NTRK2* or *NTRK3* rearrangements. *ALK* rearrangements were the most common, with 48 (23%) cases defined as positive: 43 cases showed an *ALK* split signal in ≥50% of the analyzed neoplastic cells, and in the remaining 5 cases, *ALK* rearrangement was observed in percentages varying from 15% to 40%. The most common observed *ALK* fusion was *EML4::ALK* (38 cases, Figure 1), identified by the three-color *EML4-ALK* FISH probe (green 5′ and red 3′ *ALK* split signals, each of them fused with the split aqua *EML4* signal) (Figure 1A,B); the hybridization pattern observed in the remaining 10 cases suggested an *ALK* rearrangement not involving *EML4* (green 5′ and red 3′ *ALK* split signals, without aqua *EML4* signal involvement). Reflex ALK IHC was performed in all the 48 *ALK* FISH-positive cases: all the cases showed strong cytoplasmic immunoreactivity and were scored as ALK-positive (Figure 1C). 

In one case (Pt ID 186), we observed only rare cells (8%) with a green 5′ and red 3′ *ALK* split signal and *EML4* involvement. This observation was associated with weak ALK cytoplasmatic immunoreactivity; therefore, the case was defined as *ALK*-negative.

ALK protein expression was not detected in the remaining 162 cases of the study cohort, which were all scored as ALK IHC-negative .

In total, 19 cases (9%) showed a red 5′ and green 3′ *ROS1* split signal by FISH: 17 cases in ≥50% of the analyzed neoplastic cells and, in the remaining 2 cases, in 25% and 26%, respectively. 

In 8 cases (4.1%), a red 5′ and green 3′ *RET* FISH split signal was observed in ≥50% of the tumor cells, while 1 case showed a single green 3′ *RET* signal, which was interpreted as positive according to the guidelines [14].

One case showed a green 5′ and red 3′ *NTRK3* split signal in ≥50% of the tumor cells; therefore, it was defined as *NTRK*-positive, and it has been reported elsewhere [15].

### 3.2. Fusion Variant Detection by NGS and Real-Time PCR: Molecular Approach

The two RNA-based NGS fusion panels used in this study can detect the majority of NSCLC-targetable fusion genes in an accurate and efficient manner. We succeeded in analyzing a total of 200 NSCLCs out of the 210 cohort cases, with a high success rate (95%). Ten cases did not meet the used panel quality control and were thus considered failed for NGS analysis. The NGS Fusion Sample Quality Control check failed (the total mapped fusion panel reads ≤ 20,000) because of the poor quality and quantity of the total RNA sample. In fact, for 8 samples, only very small tissue biopsies (<500 neoplastic cells) were available; the other 2 samples had been exposed to prolonged formalin fixation (>72 h). These data emphasize the crucial importance of proper tumor sampling and fixation processing, which can have a negative impact on molecular analysis if not correctly carried out. 

Among the 10 cases inadequate for NGS analysis, 3 were *ALK* FISH-positive without *EML4* involvement, and for those, ALK IHC was scored as positive; the other 7 cases did not show any rearrangement in the *ALK*, *ROS1*, *RET*, *NTRK1*, *NTRK2* or *NTRK3* genes.

The targeted RNA NGS analysis identified the presence of a fusion in 72 out of the 200 (36%) successfully analyzed cases. In particular, 38 *ALK* fusions (35 cases with *EML4* as the partner gene and 3 cases with *KIF5B*), 14 *ROS1* fusions (5 cases involving *CD74*, 4 cases involving *SDC4*, 4 cases involving *SLC34A2* and 1 case involving *EZR*), 5 *RET* fusions (all of them having *KIF5B* as the partner gene) and 1 *NTRK* fusion (*EML4(2)::NTRK3(14)*) were identified. 

In addition to the *ALK*, *ROS1*, *RET*, *NTRK1*, *NTRK2* and *NTRK3* fusions, the used NGS panels can detect a wide range of gene alterations. This extended analysis allowed us to identify 12 cases carrying *MET* exon 14 skipping, as well as 2 other gene fusions (*EIF3E(1)::RSPO2(2)* and *FGFR3(17)::TACC3(10)*).

We analyzed 198 NGS-tested samples also by RT-PCR (the 2 cases carrying gene fusions not involving *ALK*, *ROS1*, *RET*, *NTRK1*, *NTRK2* or *NTRK3* were excluded because those genes are not detected by the RT-PCR kit panel). We identified the presence of an alteration in 71 cases (36%): 39 *ALK* fusions, 14 *ROS1* fusions, 5 *RET* fusions, 1 *NTRK* fusion and 12 *MET* exon 14 skipping. Three discordant cases were observed: two NGS-negative cases tested positive for *ALK* fusion by RT-PCR, and one case with an ALK fusion detected by NGS tested negative by RT-PCR. This last *ALK* fusion was not included in the RT-PCR kit panel. 

**Figure 2:**
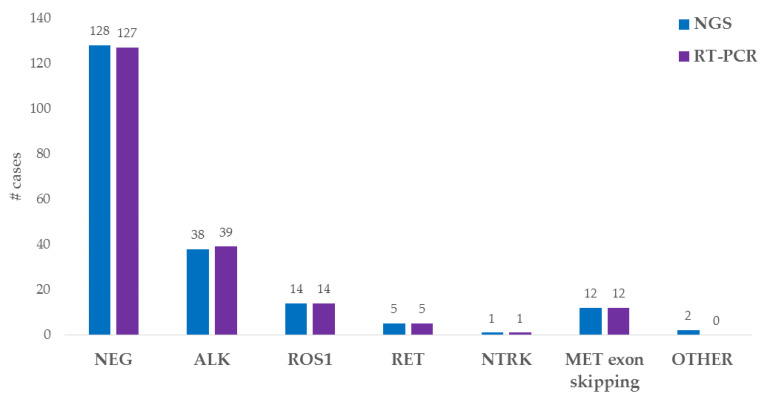
Histogram representing NGS and RT-PCR results.

The NGS and RT-PCR data are summarized in Figure 2.

### 3.3. Comparison of the In Situ and Molecular Approaches

Concordant results were observed in 182 cases (91%) out of the 200 analyzed by both FISH and NGS. In particular, 57 cases resulted positive using both approaches, showing *ALK* (37), *ROS1* (14), *RET* (5) or *NTRK* (1) rearrangements (Table 1). The other 125 concordant cases included 111 *ALK/ROS1/RET/NTRK* FISH-negative and NGS-negative cases for all the targeted fusions and 14 cases that were NGS-positive for a fusion involving genes other than *ALK*, *ROS1*, *RET*, *NTRK1*, *NTRK2* or *NTRK3* (i.e., 12 *MET* exon 14 skipping, *EIF3E::RSPO2* and *FGFR3::TACC3*).

A total of 198 cases were analyzed by both FISH and RT-PCR for *ALK/ROS1/RET/NTRK*, and we observed 181 (91.4%) concordant cases, with 58 and 123 cases being positive and negative, respectively, by the two methods (Table 1).

Discordant results between FISH and NGS were found in 18 cases (9%) (Table 2): 17 (94%) targeted RNA NGS-negative cases had a positive result by FISH, and 1 (6 %) case positive for a fusion by NGS had a FISH-negative result. In particular, 13 NGS-negative cases presented gene rearrangement in ≥50% of the analyzed cells by FISH (6 *ALK* fusions, 3 *ROS1* fusions and 4 *RET* fusions), and 4 cases showed 20–25% of rearranged cells by FISH (2 *ALK* and 2 *ROS1* rearrangements). 

According to the previously published studies [2], all the FISH *ALK*-positive cases showed ALK positivity by reflex IHC.

The RT-PCR data were in line with the NGS results, but we observed 17 (8.6%) cases that were discordant with the FISH results. In fact, 16 FISH-positive samples did not show any gene fusions by RT-PCR analysis.

One case (Pt ID186) showed *ALK* split signals in rare cells (8%) by FISH and had been therefore scored negative because this value was below the cut-off of positivity (i.e., 15%); nevertheless, both NGS and RT-PCR analyses could identify an *ALK* fusion. 

### 3.4. Selected Patients’ Clinical Follow-Up

A total of five cases with discordant in situ/NGS results and sufficient clinical data after initiation of TKI therapy were identified (cases 182, 183, 184, 190 and 194). Two FISH-*ALK*-rearranged patients (Pt ID 182 and Pt ID 183) treated with Alectinib did not develop progression after 24 and 12 months, respectively. One FISH-*ALK*-rearranged (Pt ID184) and one FISH-*ROS1*-rearranged (Pt ID194) patients received Crizotinib as first-line therapy, and they progressed after 6 and 18 months, respectively. The FISH-*ALK*-rearranged patient was then treated with Ceritinib, experienced progression after 22 months, received Brigatinib as third-line therapy and died of the disease after 12 months. The FISH-*ROS1*-rearranged patient showed response to Lorlatinib, given as second-line therapy, at 24-month follow-up. One FISH-*RET*-rearranged patient had progression on Selpercatinib at 6 months and died of the disease after another 6 months. 

## 4. Discussion

Nowadays, testing for gene fusions is essential to identify NSCLC patients who can benefit from personalized, targeted treatment. Though in the first instance international and national guidelines for advanced LuAds [16] recommend only *ALK* and *ROS1* testing, an updated and efficient molecular diagnostics of LuAds should not exclude the simultaneous analysis of all the targetable gene fusions, as there are already approved targeted therapies that can be employed for selected patients. Importantly, a wide-ranging molecular analysis should be performed as soon as possible at the diagnosis of stage IV LuADs in order to guarantee the patient the most appropriate available treatment in a rapid and cost-efficient manner.

While the low percentage of RTK fusions observed in LuADS [4] may discourage an initial comprehensive analysis of all of them, the identification of these low-prevalence alterations, especially in younger, non-smoking patients, can play a key role in their cure [15]. In this study, we demonstrated how, in a clinical setting, a comprehensive multiplexing approach for gene fusion detection that simultaneously queries multiple potentially actionable targets (e.g., *ALK*, *ROS1*, *RET*, *NTRK1*, *NTRK2*, *NTRK3* and *MET ex14skipping*) is feasible by targeted NGS using total RNA extracted from FFPE NSCLC clinical samples. We analyzed a total of 210 NSCLC samples by FISH, and we succeeded in characterizing 200 of these samples (95%) by targeted RNA NGS and 198 samples (94%) by RT-PCR. The results of our analyses, together with already published data [17,18,19,20], showed an excellent concordance rate (91%) of the targeted RNA NGS results with the so-far considered gold standard FISH technique, underlining the essential role of the NGS testing approach in the molecular pathology diagnostics laboratory.

Nevertheless, the minimal discordance (9%) found in the comparison of the FISH results with the NGS and RT-PCR data prompts thoughts and suggests the need for careful planning of diagnostic strategies.

Any targeted molecular assay (both NGS and RT-PCR) is intrinsically limited by the fact that it cannot detect gene fusions that are not present in the panel design used, which is commonly based on the literature’s most recent evidence. Although the most frequent targetable gene fusions are usually included in the commercially available kits, rare and unknown fusions could be missed by these approaches, especially for the less frequent and therefore poorly studied *RET* and *NTRK1*, *NTRK2* and *NTRK3* fusions. 

In recent years, Anchored Multiplex PCR (AMP) RNA NGS assays, based on the RACE (Rapid Amplification of cDNA Ends) PCR principle, have been developed [21] and successfully employed in the identification of gene rearrangements without prior knowledge of the target gene fusion partners [22,23]. This approach can be definitely advantageous when analyzing promiscuous gene fusions, such as those involving RTK genes in LuADs, though it requires higher RNA quantities (50–200 ng) and, therefore, cannot be always applicable.

We successfully demonstrated that, in those targeted RNA NGS/RT-PCR-negative cases, ALK IHC and FISH analyses using break-apart *ALK*, *ROS1*, *RET*, *NTRK1*, *NTRK2* and *NTRK3* probes did help to unveil the presence of gene fusions. While a FISH split signal for these genes cannot indicate whether the rearrangement results in an in-frame functional gene fusion, it may still identify a targetable alteration, especially in those non-smoking LuAD patients who do not carry any other targetable molecular alteration. In the case of FISH ALK split signals, ALK IHC successfully demonstrated the protein overexpression and its localization, therefore confirming the significance of a FISH ALK-positive result when NGS data are negative for the presence of a gene fusion. In fact, ALK IHC has been approved as a stand-alone companion diagnostic test [2].

Other less common rearrangements unveiled by a positive FISH finding could be further studied with other methods, such as AMP-PCR RNA NGS and RNAseq, in order to establish their role as a target for a specific drug. 

For the 17 FISH-positive and NGS-negative discordant cases, we can hypothesize different explanations. The discordance may be due to the presence of a gene fusion different from those covered by the NGS and RT-PCR panels used. In fact, targeted approaches investigate only specific genome regions of interest with selected primers of amplification, which do not cover all the possible gene rearrangements. Further studies are needed in order to identify the gene breakpoints and compare those gene fusions to the ones included in the NGS and RT-PCR panel designs. For the cases that showed a FISH split signal with a percentage of tumor cells lower than 50%, we may also think of a too low representation of the altered cells and, therefore, a limited sensitivity of the NGS technique. On the contrary, the discordant FISH-negative NGS-positive case, which showed a low percentage of *ALK* split signals by FISH (8%) and an *EML4::ALK* NGS/RT-PCR-identified fusion, would indicate a high sensitivity of both methods. Moreover, this discrepancy would also suggest the importance of a more careful application of the FISH interpretation guidelines cut-off for *ALK* and other RTKs, which is ≥15%.

In our study cohort, the significance of the FISH-positive and NGS/RT-PCR-negative discordant findings was ultimately corroborated by the patients’ responses to targeted therapy.All three ALK FISH-positive and ALK IHC-positive cases clinically responded to the ALK-targeted treatment, and two of them are still alive after 24 months and 12 months, respectively, while the third one died of the disease after 40 months following three lines of targeted treatment. Though the presented cases are very few and anecdotal, they still indicate the strength of the FISH data and suggest the key utility of the method in those LuAD cases that tested fusion-negative by targeted RNA NGS/RT-PCR.

The availability of different testing methods as alternatives to targeted RNA NGS assays is also decisive for those samples in which the NGS assays failed due to technical problems such as poor RNA quality and/or quantity extracted from the FFPE samples. In fact, formalin fixation can negatively affect nucleic acid quality by inducing both fragmentation and sequence artifacts that may impact the downstream assay performance, especially when RNA analysis for gene fusion detection is requested, therefore negatively affecting the possibility to obtain useful molecular data [11]. Moreover, tumor molecular characterization may also be challenging because of the lack of adequate tumor cellularity in the tissue sample: very small biopsies are often obtained in LuAD patients, limiting the amount of tissue available for tumor molecular characterization. For those low-quality and very limited samples, in situ approaches such as ALK IHC and *ALK/ROS1/RET/NTRK* FISH, which can also evaluate a small number of tumor cells, can be useful options.

## 5. Conclusions

Our experience demonstrated that the targeted RNA NGS characterization of LuADs allows accurate RTK fusion detection; therefore, it should be routinely performed in efficient molecular pathology diagnostic laboratories. Nevertheless, standard methods such as IHC and FISH should not be dismissed and completely replaced by NGS techniques. Currently, they are definitely cost-effective compared to the costs of molecular assays, and, though they are not used upfront, they should still be available as they can crucially contribute to completing the molecular characterization of LuADs, either when NGS cannot be performed because of limited tissue availability or when no molecular targetable alterations are found, especially in younger and non-smoking patients.

## Figures and Tables

**Table 1:** FISH-Positive/NGS-Positive Concordant Cases.

No	Pt_ID	FISH	NGS	RT-PCR	ALK IHC	FISH_Data	NGS_Data	RT-PCR_Data
1	2	+	+	+	+	*ALK* split >50%, no *EML4*	*KIF5B(24)::ALK(20)*	*ALK* POS
2	3	+	+	+	+	*ALK* split >50%, no *EML4*	*KIF5B(17)::ALK(20)*	*ALK* POS
3	4	+	+	+	+	*ALK* split >50%, no *EML4*	*KIF5B(17)::ALK(20)*	*ALK* POS
4	5	+	+	+	+	*EML4::ALK* > 50%	*EML4(20)::ALK(20)*	*ALK* POS
5	6	+	+	+	+	*EML4::ALK* > 50%	*EML4(6)::ALK(20)*	*ALK* POS
6	7	+	+	+	+	*EML4::ALK* > 50%	*EML4(6)::ALK(20)*	*ALK* POS
7	8	+	+	+	+	*EML4::ALK* > 50%	*EML4(6)::ALK(20)*	*ALK* POS
8	9	+	+	+	+	*EML4::ALK* > 50%	*EML4(6)::ALK(20)*	*ALK* POS
9	10	+	+	+	+	*EML4::ALK* > 50%	*EML4(2)::ALK(20)*	*ALK* POS
10	11	+	+	+	+	*EML4::ALK* > 50%	*EML4(13)::ALK(20)*	*ALK* POS
11	12	+	+	+	+	*EML4::ALK* > 50%	*EML4(13)::ALK(20)*	*ALK* POS
12	13	+	+	+	+	*EML4::ALK* > 50%	*EML4(13)::ALK(20)*	*ALK* POS
13	14	+	+	-	+	*EML4::ALK* > 50%	*EML4(6)::ALK(18)*	NEG
14	15	+	+	+	+	*EML4::ALK* > 50%	*EML4(13)::ALK(20)*	*ALK* POS
15	16	+	+	+	+	*EML4::ALK* > 50%	*EML4(13)::ALK(20)*	*ALK* POS
16	17	+	+	+	+	*EML4::ALK* > 50%	*EML4(20)::ALK(20)*	*ALK* POS
17	18	+	+	+	+	*EML4::ALK* > 50%	*EML4(13)::ALK(20)*	*ALK* POS
18	19	+	+	+	+	*EML4::ALK* > 50%	*EML4(20)::ALK(20)*	*ALK* POS
19	20	+	+	+	+	*EML4::ALK* > 50%	*EML4(6)::ALK(20)*	*ALK* POS
20	21	+	+	+	+	*EML4::ALK* > 50%	*EML4(13)::ALK(20)*	*ALK* POS
21	22	+	+	+	+	*EML4::ALK* > 50%	*EML4(6)::ALK(20)*	*ALK* POS
22	23	+	+	+	+	*EML4::ALK* >50%	*EML4(6)::ALK(20)*	*ALK* POS
23	24	+	+	+	+	*EML4::ALK* > 50%	*EML4(13)::ALK(20)*	*ALK* POS
24	25	+	+	+	+	*EML4::ALK* > 50%	*EML4(13)::ALK(20)*	*ALK* POS
25	26	+	+	+	+	*EML4::ALK* > 50%	*EML4(6)::ALK(20)*	*ALK* POS
26	27	+	+	+	+	*EML4::ALK* > 50%	*EML4(13)::ALK(20)*	*ALK* POS
27	28	+	+	+	+	*EML4::ALK* > 50%	*EML4(13)::ALK(20)*	*ALK* POS
28	29	+	+	+	+	*EML4::ALK* > 50%	*EML4(13)::ALK(20)*	*ALK* POS
29	30	+	+	+	+	*EML4::ALK* > 50%	*EML4(13)::ALK(20)*	*ALK* POS
30	31	+	+	+	+	*EML4::ALK* > 50%	*EML4(2)::ALK(20)*	*ALK* POS
31	32	+	+	+	+	*EML4::ALK* > 50%	*EML4(20)::ALK(20)*	*ALK* POS
32	33	+	+	+	+	*EML4::ALK* > 50%	*EML4(6)::ALK(20)*	*ALK* POS
33	34	+	+	+	+	*EML4::ALK* > 50%	*EML4(6)::ALK(20)*	*ALK* POS
34	35	+	+	+	+	*EML4::ALK* > 50%	*EML4(13)::ALK(20)*	*ALK* POS
35	212	+	+	+	+	*EML4::ALK* > 50%	*EML4(6)::ALK(20)*	*ALK* POS
36	36	+	+	+	+	*EML4::ALK* = 12%	*EML4(6)::ALK(20)*	*ALK* POS
37	37	+	+	+	+	*EML4::ALK* = 40%	*EML4(13)::ALK(20)*	*ALK* POS
38	162	+	+	+	-	*EML4::NTRK3* > 50%	*EML4(2)::NTRK3(14)*	*NTRK* POS
39	166	+	+	+	-	*ROS1* split > 50%	*SDC4(2)::ROS1(32)*	*ROS1* POS
40	167	+	+	+	-	*ROS1* split > 50%	*SLC34A2(13)::ROS1(34)*	*ROS1* POS
41	168	+	+	+	-	*ROS1* split > 50%	*EZR(10)::ROS1(34)*	*ROS1* POS
42	169	+	+	+	-	*ROS1* split > 50%	*CD74(6)::ROS1(34)*	*ROS1* POS
43	170	+	+	+	-	*ROS1* split > 50%	*SLC34A2(13)::ROS1(32)*	*ROS1* POS
44	171	+	+	+	-	*ROS1* split > 50%	*SDC4(2)::ROS1(32)*	*ROS1* POS
45	172	+	+	+	-	*ROS1* split > 50%	*CD74(6)::ROS1(34)*	*ROS1* POS
46	173	+	+	+	-	*ROS1* split > 50%	*CD74(6)::ROS1(34)*	*ROS1* POS
47	174	+	+	+	-	*ROS1* split > 50%	*SLC34A2(13)::ROS1(32)*	*ROS1* POS
48	175	+	+	+	-	*ROS1* split > 50%	*SLC34A2(13)::ROS1(32)*	*ROS1* POS
49	176	+	+	+	-	*ROS1* split > 50%	*SDC4(2)::ROS1(32)*	*ROS1* POS
50	177	+	+	+	-	*ROS1* split > 50%	*SDC4(2)::ROS1(32)*	*ROS1* POS
51	209	+	+	+	-	*ROS1* split > 50%	*CD74(6)::ROS1(34)*	*ROS1* POS
52	210	+	+	+	-	*ROS1* split > 50%	*CD74(6)::ROS1(34)*	*ROS1* POS
53	163	+	+	+	-	*RET* split > 50%	*KIF5B(15)::RET(12)*	*RET* POS
54	164	+	+	+	-	*RET* split > 50%	*KIF5B(15)::RET(12)*	*RET* POS
55	165	+	+	+	-	*RET* split > 50%	*KIF5B(15)::RET(12)*	*RET* POS
56	207	+	+	+	-	*RET* split > 50%	*KIF5B(23)::RET(12)*	*RET* POS
57	208	+	+	+	-	*RET* split > 50%	*KIF5B(15)::RET(12)*	*RET* POS

Abbreviations: No, Number; +, positive for presence of rearrangement/expression; -, negative for presence of rearrangement/expression.

**Table 2:** FISH and NGS/RT-PCR Discordant Cases.

No	Pt_ID	FISH	NGS	RT-PCR	ALK IHC	FISH_Data	NGS_Data	RT-PCR_Data
1	178	+	-	-	+	*ALK* split = 25%, no *EML4*	No fusions	NEG
2	179	+	-	-	+	*ALK* split = 25%, no *EML4*	No fusions	NEG
3	180	+	-	-	+	*ALK* split = 50%, no *EML4*	No fusions	NEG
4	181	+	-	-	+	*ALK* split = 50%, no *EML4*	No fusions	NEG
5	182	+	-	+	+	*EML4::ALK* > 50%	No fusions	*ALK* POS
6	183	+	-	-	+	*EML4::ALK* > 50%	No fusions	NEG
7	184	+	-	-	+	*EML4::ALK* > 50%	No fusions	NEG
8	185	+	-	+	+	*EML4::ALK* > 50%	No fusions	*ALK* POS
9	192	+	-	-	-	*ROS1* split > 50%	No fusions	NEG
10	193	+	-	-	-	*ROS1* split > 50%	No fusions	NEG
11	194	+	-	-	-	*ROS1* split > 50%	No fusions	NEG
12	195	+	-	-	-	*ROS1* split = 25%	No fusions	NEG
13	196	+	-	-	-	*ROS1* split = 25%	No fusions	NEG
14	189	+	-	-	-	*RET* split > 50%	No fusions	NEG
15	190	+	-	-	-	*RET* split > 50%	No fusions	NEG
16	191	+	-	-	-	*RET* split > 50%	No fusions	NEG
17	188	+	-	-	-	*RET* single 3′ > 50%	No fusions	NEG
18	186	-	+	+	-	*ALK* split in rare cell	*EML4(6)::ALK(20)*	*ALK* POS

Abbreviations: No, Number; +, positive for presence of rearrangement/expression; -, negative for presence of rearrangement/expression.

## Data Availability

The data presented in this study are available upon request from the corresponding author.
